# Nanosized hydroxyapatite and β-tricalcium phosphate composite: Physico-chemical, cytotoxicity, morphological properties and *in vivo* trial

**DOI:** 10.1038/s41598-019-56124-4

**Published:** 2019-12-20

**Authors:** Igor da Silva Brum, Jorge José de Carvalho, Jorge Luis da Silva Pires, Marco Antonio Alencar de Carvalho, Luiza Braga Ferreira dos Santos, Carlos Nelson Elias

**Affiliations:** 1 Universidade do Estado do Rio de Janeiro, Rio de Janeiro, Brazil; 2 Instituto Militar de Engenharia, Rio de Janeiro, Brazil

**Keywords:** Biomedical engineering, Biomedical materials, Nanoparticles

## Abstract

The objective of this work was to characterize the properties of a synthetic biomaterial composite with nanoparticles size (Blue Bone). This biomaterial is a composite recommended for dental and orthopedic grafting surgery, for guided bone regeneration, including maxillary sinus lift, fresh alveolus filling, and treatment of furcation lesions. The nano biomaterials surface area is from 30% to 50% higher than those with micro dimensions. Another advantage is that the alloplastic biomaterial has homogeneous properties due to the complete manufacturing control. The analyzed biomaterial composite was characterized by XRD, cytochemistry, scanning electron microscopy, porosimetry and *in vivo* experiments (animals). The results showed that the analyzed biomaterial composite has 78.76% hydroxyapatite [Ca_5_(PO_4_)_3_(OH)] with monoclinic structure, 21.03% β-tricalcium phosphate [β -Ca_3_(PO_4_)_2_] with trigonal structure and 0.19% of CaO with cubic structure, nanoparticles with homogeneous shapes, and nanoporosity. The *in vivo* experiments showed that the composite has null cytotoxicity, and the site of insertion biomaterials has a high level of vascularization and bone formation. The conclusion is that the synthetic biomaterial with Blue Bone designation presents characteristics suitable for use in grafting surgery applications.

## Introduction

Several techniques are used in the practice of dental and orthopedic surgery to increase hard tissue volume. Historically, the best results were obtained with autogenous bone grafts, which have a great capacity of revascularization^1^. This type of bone graft has the disadvantage of requiring donor surgery for bone collection. Among the possible regions for collecting the patient’s bone, there are mandibular symphysis, calvaria, mandible, zygomatic bone, and ribs. This kind of intervention may present high morbidity during the surgical procedure. To eliminate these drawbacks, homogeneous, heterogeneous and alloplastic grafts are employed.

As Olszta *et al*.^1^, bone is a hierarchically structured composite material that due to its unique microstructure and mechanical properties has a high biologically value. Secondary bone (osteonal) is a laminated organic-inorganic compound composed mainly of collagen, hydroxyapatite, and water. Hydroxyapatite (HA) is a biomaterial widely used in orthopedic and dental surgery as it exhibits the ability to promote bone growth. *In vivo* studies analyzed differences in the numbers of osteocytes and osteoblasts in areas grafted with synthetic biomaterial composite of 70% hydroxyapatite and 30% calcium beta-phosphate, concluding that there was no statistical difference between groups when compared with native bone^2^. Despite some deleterious effects, there is a trend towards the use of synthetic biomaterials in bone grafts instead of natural ones^3^. Among the various synthetic biomaterials, hydroxyapatite gives the best results. In the market of dental implantology and orthopedic are offered several types of hydroxyapatite. Nanosized hydroxyapatite particles have a larger surface area than micrometric particles^4^. Also, the results of *in vitro* tests (cell culture) and *in vivo* tests (surgical implantation) show that hydroxyapatite nanoparticles have excellent biocompatibility^5,6^.

Results from the literature indicate that, a few months after grafting surgery, synthetic hydroxyapatite regenerates the bone^7^. The results with micro-CT (μ-CT) confirmed histological data. For predictable results, nanohydroxyapatites should be comparable to natural bone grafts used for bone regeneration.

Godoy *et al*.^8^ evaluated and monitored the radiographic results of dental implant installation surgeries at critical sites where hydroxyapatite grafting was performed. Clinical results showed that implant osseointegration occurred without complications, indicating that with the use of a good surgical technique, the risk of osseointegration failure is minimal or absent.

The objective of the present work was to analyze and characterize the morphology, physical, chemical and cytotoxic properties of a new nanosized composite of hydroxyapatite and β-tricalcium phosphate. The difference between the analyzed composite in the present work and those already in the market is the use of new techniques of synthesis of biomaterials. The studied synthetic biomaterial has particles nano-sized, and to prepare the putty, it’s not necessary to mix it with the patient’s blood. The composite is ready to use after a mixture with sterile saline.

## Materials and Methods

Composite samples of nanometric particles of hydroxyapatite and β-tricalcium phosphate, named as Blue Bone were synthesized in the present work. *In vitro* test was used to characterize the properties of the biomaterials and *in vivo* experimental with rat model were done to analyze the biomaterial biocompatibility.

The biomaterial was characterized by X-ray diffraction (XRD), porosimetry and pycnometer tests. The morphology of the samples was characterized using scanning electron microscopy (SEM) Field Emission Gun (Quanta FEG 250; Hillsboro, Oregon 97124 - USA).

The X-ray diffraction (XRD) was performed using a Panalytical (Almelo, Netherlands) Empyrean diffractometer, with Cu-Kα radiation, 2θ range of 20–80°, a step width of 0.02°, and an exposure time of 5 s.

The diffraction peaks were identified based on comparison with standard ICDD (International Centre for Diffraction Data) diffraction files and COD-Jan2012 (Crystallography Open Database) PDF2-2004 databases. The Rietveld method was employed to quantify the phases and to analyze the crystal structures. The following diffraction files were used:

Ca5 (PO4) 3 (OH): diffraction file # 01-086-0740

β-Ca3 (PO4) 2: diffraction file # 00-009-0169

Ca5 (PO4) 3 (OH): diffraction file # 01-076-0694

CaO: diffraction file # 01-082-1691

Rietveld analysis of XRD data was used to identify and quantify the percentages of the phases. The X-ray diffractograms were recorded on a Siemens diffractor (Bruker AXS; Durham - UK), model D-5000 (θ-θ), equipped with graphite curved monochromator, secondary beam, Cu tube. The quantitative analysis of the phases was determined by the mathematical refinement method proposed by Rietveld.

The Rietveld Method consists of adjusting the theoretical diffraction peaks, calculated from crystallographic information to the experimentally measured diffraction pattern. The criterion for this adjustment is to minimize the sum of the squares of the differences between the calculated and observed counts in the measured angular range.

1 $${S}_{Y}={\sum }_{i}{w}_{i}{({y}_{obs}-{y}_{cale})}^{2}$$

where:

i) ${w}_{i}=\frac{1}{\sqrt{{y}_{obs}}}$ it’s a weight function;
ii) y_obs_ is the count observed in the ith step;
iii) y_cal_ the calculated count in the i-th step

The quantity y_obs_ is obtained directly from the data collected on the diffractometer. The quantity y_cale_ can be calculated as:

2 $${{\rm{Y}}}_{{\rm{cale}}}={{\rm{S}}}_{{\rm{R}}}\,{\sum }_{{\rm{p}}}{{\rm{S}}}_{{\rm{p}}}\,{\rm{Ab}}\,{\sum }_{{\rm{k}}}{|{\rm{F}}({\rm{hkl}})|}^{2}\ominus ({2}_{\ominus {\rm{i}}}-{2}_{\ominus {\rm{k}}})\,{{\rm{AsL}}}_{{\rm{k}}}{{\rm{P}}}_{{\rm{k}}}+{{\rm{y}}}_{{\rm{bi}}}$$

where:

i) S_R_ is a function used to adjust the effects of surface roughness, as this is a characteristic of the sample and not of each phase and is out of the sum;
ii) S_p_ is the scale factor for the phase “p”;
iii) Ab is an absorption factor, which in the case of measurement using a Bragg-Brentan geometry, corresponds to the inverse of the sample absorption coefficient;
iv) F(hkl) is the structure factor;
v) Ѳ (2_Ѳi_ − 2_Ѳk_) is the profile function that approximates the effects of instrumental and sample characteristics;
vi) As is a function of profile asymmetry;
vii) L_k_ contains Lorentz and polarization factors;
viii) P_k_ is a preferred orientation function;
ix) y_bi_ is the background radiation contribution.

In a Rietveld analysis, there is uncertainty about y_obs_, as called standard uncertainty. The true meaning of σ[y_obs_] is that one can know the true value of the coce by a measurement is done infinite times of y_obs_, so that y_cale_ = y_obs_ + σ[y_obs_]

However, the uncertainty associated with the value of y_obs_ is calculated using the equation

σ^2^ [y_obs_] = (y_obs_ − (y_obs_))^2^, where y_obs_ is the average value of y_obs_. When intensities are measured directly by individual photon or neutron counts in the detector, the expected value is reduced to σ^2^ [y_obs_] = y_obs_, that is, the standard uncertainty depends on the square root of the observed count. The minimize function is weighted by the weight function w2 and the function in this case, is the inverse of the standard uncertainty: ${w}_{i}=\frac{1}{{\sigma }^{2}[{y}_{obs}]}$.

The R_wp_ indices, which evaluates the quality of refinement by calculating the percentage difference from the calculated and observed point to point count as:

3 $${R}_{wp}^{2}=\frac{{\sum }_{i}{w}_{i}{({y}_{obs}-{y}_{cal})}^{2}}{{\sum }_{i}{w}_{i}{({y}_{obs})}^{2}}$$

In an ideal model, the average value of (y_obs_ − y_cal_)^2^ can be equal to σ^2^[y_obs_] and the expected value of w_i_ (y_obs_ − y_cal_)^2^ equals one. In this case, what could be obtained with this ideal model is the best possible value that can ever be obtained for a real data set. The best possible value for R_wp_ is designated as R-waited, or just R_wait_ given as:

4 $${R}_{wait}^{2}=\frac{N}{{\sum }_{i}{w}_{i}{({y}_{obs})}^{2}}$$

where, $N$ is the number of points calculated.

From the magnitudes R_wp_ and R_wair_, a refinement quality factor, also known as “goodness-of-fit” or simply GOF, given as:

5 $$s=\frac{{R}_{wp}}{{R}_{wait}}$$

The true porosity of the biomaterial was calculated by the gas pycnometer technique from the measured drop in pressure when a known volume of gas is allowed to expand into a chamber containing the sample. Usually, helium is used because this gas, in addition to being inert, penetrates easily into the pores (accessible) of the sample, due to the small size of its atoms, thus allowing more accurate measurements. The helium pycnometer consists of two chambers of known volume. The first chamber is where the sample is placed and the second chamber is the expansion chamber, both chambers are connected by an expansion valve. Before starting the analysis, a degassing process is carried out, which consists of repeated vacuum and purges with helium to remove impurities and moisture. The whole system is then brought to atmospheric pressure, the expansion valve is closed and the chamber containing the sample is brought to a pressure of the order of 17 psi (P1). The expansion valve is then opened and closed several times to reduce the pressure to P2 value.

Considering helium as an ideal gas, the volume of the solid is calculated from equation:

6 $${\rm{P}}1=({\rm{Va}}-{\rm{Vs}})={\rm{P}}2({\rm{Va}}-{\rm{Vs}}+{\rm{Ve}})$$

where Va is the volume of the chamber equipment, Vs is the sample volume and Ve is the volume of the expansion chamber. The main advantage of this method lies in the possibility of measuring only the solid volume of the substance, i.e., one can eliminate the pores from the sample volume. This method is suitable for samples with open pores that allow gas diffusion. Moreover, it allows, in principle, to measure volumes of solids with any moisture content.

The method of mercury intrusion porosimetry is based on the principle that the intrusion of mercury under pressure is controlled by the pore diameter through the Laplace equation:

7 $$d=-\,4.{\rm{\gamma }}.\,\cos \,{\rm{\theta }}\,3{\rm{P}}$$

where γ is the interfacial tension between mercury and air (0.48 N/m), P is the applied pressure, θ is the contact angle and d is the pore diameter.

The total porosity was determined by Eq. 8:

8 $$P=\frac{VMP}{VMP+Ma/\rho }$$

where P is the total porosity, VMP is the volume of intruded mercury, Ma is the mass of the sample and ρ is the density of the sample.

Cytotoxicity analyses were performed by the agar diffusion method using triplicates of the samples.

For cell monolayer formation, NCTC Clone 929 (mouse connective tissue cell (ATCC CCL 1) cell lines at a concentration of 3.0 × 105 cells/mL were sown in Petri dishes and incubated for 48 hours at 37 °C in a humidified incubator with an atmosphere of 5% CO_2_. The liquid culture medium was replaced by a solid cover medium (which is composed of equal parts of twice concentrated medium and 1.8% agar with 0.01% neutral red). Sample fragments (0.25 cm^2^) were placed on this cover medium before its complete solidification. The plates were again incubated at 37 °C with a 5% CO_2_ atmosphere for 24 hours. The negative control group (a group in which no response is expected) was filter paper disks (0.5 cm in diameter) of non-toxic nature. The positive control group (a group that receives treatment with a known result) was latex fragments (0.5 cm × 0.5 cm) of proven toxic nature. The samples were tested in triplicates on separate plates. The plates were analyzed by optical microscopy to determine cell integrity around the sample and macroscopically for the presence of a halo. Toxicity is observed when there is a clear halo around the sample.

For biocompatibility tests, an animal model was used. Wistar adults rats with 250 g (n = 48) were selected. In the calvaria of each animal 2 surgical sites with a diameter of 3 mm were prepared. One of the surgical sites was filled with the Bio-Oss (Geistlich Pharma Brazil, Co) biomaterial (control group) and the other was filled with Blue Bone (test group). The animals were sacrificed 4 weeks after surgery and samples tissue reactions were histologically evaluated Animals were supplied by Centro de Criação de Animais at Instituto Oswaldo Cruz (Rio de Janeiro, RJ-Brazil). This study was approved by the institutional review board and the animal ethical committee at Oswaldo Cruz Institute. The protocol number was 001-2019. Surgery details are described by Coimbra *et al*.^9^.

For the histomorphometric study, it was used the program Prism.G for Windows. In each animal calvaria sample, the region filled with Bio-Oss and the region that was inserted the Blue Bone were analyzed.

## Results

**Figure 1:**
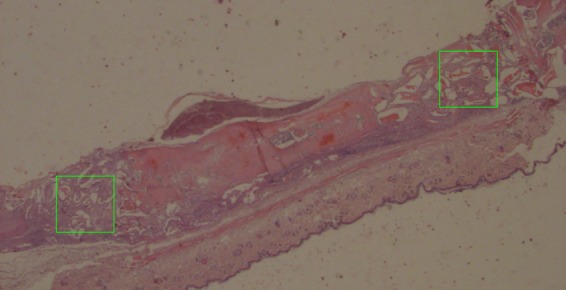
The analyzed animal calvaria sample 4 weeks after surgery. 12.5x magnification.

Figure 1 shows one analyzed sample 4 weeks after surgery. The demineralized area with biomaterials and new tissue formation area were calculated.

**Figure 2:**
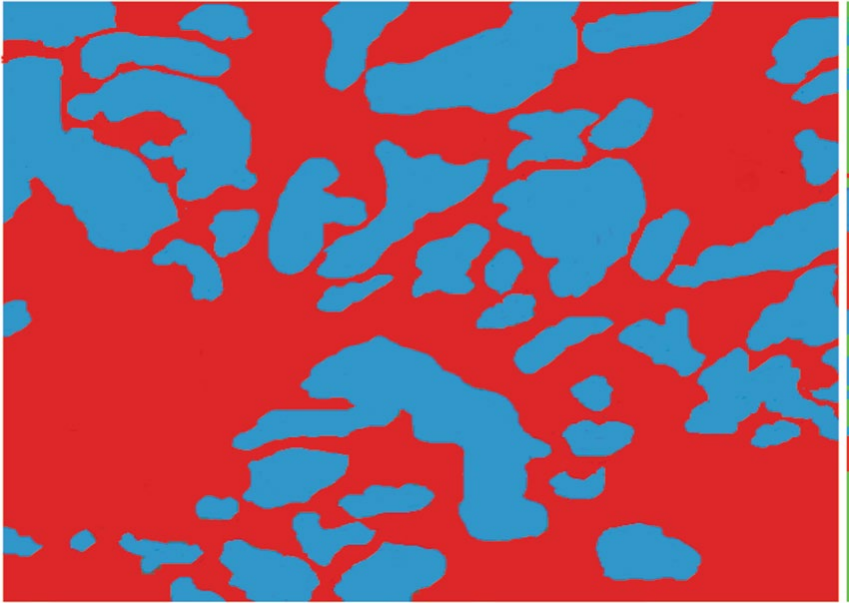
Histomorphology region with remaining unabsorbed Blue Bone biomaterial particles (blue area) and region with new tissue (red color). Sample obtained 4 weeks after surgery.

**Figure 3:**
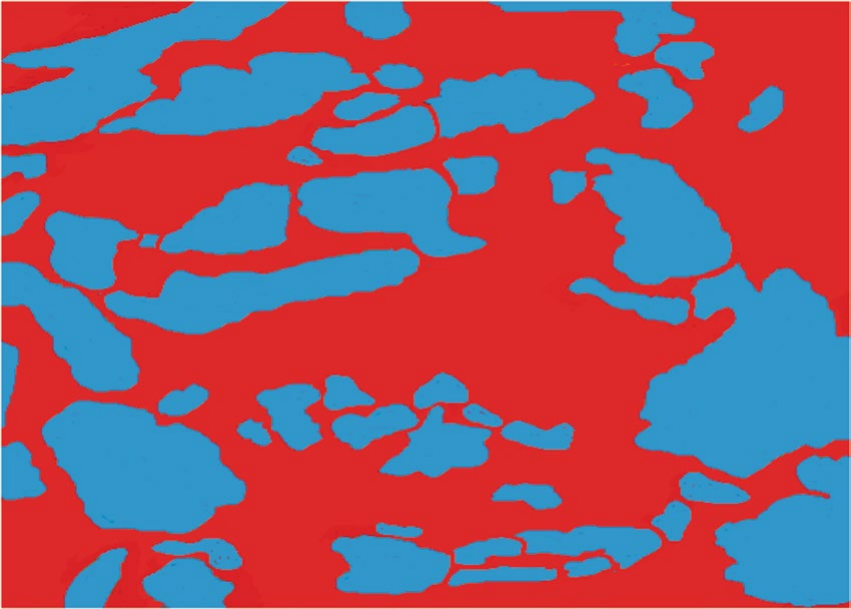
Histomorphology region with remaining unabsorbed Bio-Oss biomaterial particles (blue area) and region with new tissue (red color). Sample 4 weeks after surgery.

Figures 2 and 3 show one sample of the analyzed tissue morphologies after surgery. In these figures, the area with biomaterials has a blue color and the new tissue formation area has a red color.

Table 1 shows the composite phases identified based on indexed crystallographic data and the calculated phase percentage obtained by the Rietveld method. One can see in Table 1 that the Blue Bone samples have a higher percentage (78.76%) of the Ca_5_(PO_4_)_3_(OH) phase than β-Ca_3_(PO_4_)_2_ (21.03%).

**Table 1:** Phases, crystal structures, unit cell parameters, percentage of Blue Bone phases and refinement quality using the Rietveld method.

Identified Phases	Crystalline structure	Parameters of the unit cell	% phase (% m/m)
Ca_5_(PO_4_)_3_(OH)	Hexagonal (P63/m)	a = b = 8.1198 Å	<0.01
		c = 19.1945 Å	
		α = β = 90°	
		γ = 120°	
β-Ca_3_(PO_4_)_2_	Symmetry trigonal (R-3c)	a = b = 10.422 Å	21.03
		c = 37.3548 Å	
		α = β = 90°	
		γ = 120 °	
Ca_5_(PO_4_)_3_OH	Monoclinic (P21/b)	a = 9.4107 Å	78.76
		b = 18.8186 Å	
		c = 6.8742 Å	
		α = β = 90°	
		γ = 120.001 °	
CaO	Cubic symmetry (Fm-3m)	a = b = c = 4.8041 Å	0.19
		α = β = γ = 90°	

The diffractogram shows defined and high intensity diffraction peaks withoutamorphous phases. Table 1 shows that Blue Bone biomaterial composite has higher percentage of monoclinic hydroxyapatite (78.76%) phase than β-Ca_3_(PO_4_)_2_ (21.03%) and CaO (0.19%) phases. Typically, hydroxyapatite has a hexagonal crystal structure. Elliot *et al*.^10^ were the first research that synthetized monoclinic hydroxyapatite. Several researchers then used different methodologies to obtain monoclinic hydroxyapatite. In the present work a new methodology was used to obtain monoclinic HA at room temperature. Hochrein *et al*.^11^ analyzed the transformation of the low-temperature (monoclinic, P21/b) to the high-temperature (hexagonal, P63/m) modification of hydroxyapatite Ca_5_[(OH)(PO_4_)_3_]. Ma and Liu^12^ produced hydroxyapatite by hydrolysis of brushite crystals and identified the monoclinic phase based on electron microscopy and electron diffraction techniques. They produced monoclinic hydroxyapatite crystals at low temperature. They explained that the structural differences between hexagonal or monoclinic hydroxyapatite are very subtle.

Results of Rietiveld method analysis showed that the refinement quality was good (Rw = 6.45, Resp = 3.56 and GOF = 1.81).

**Figure 4:**
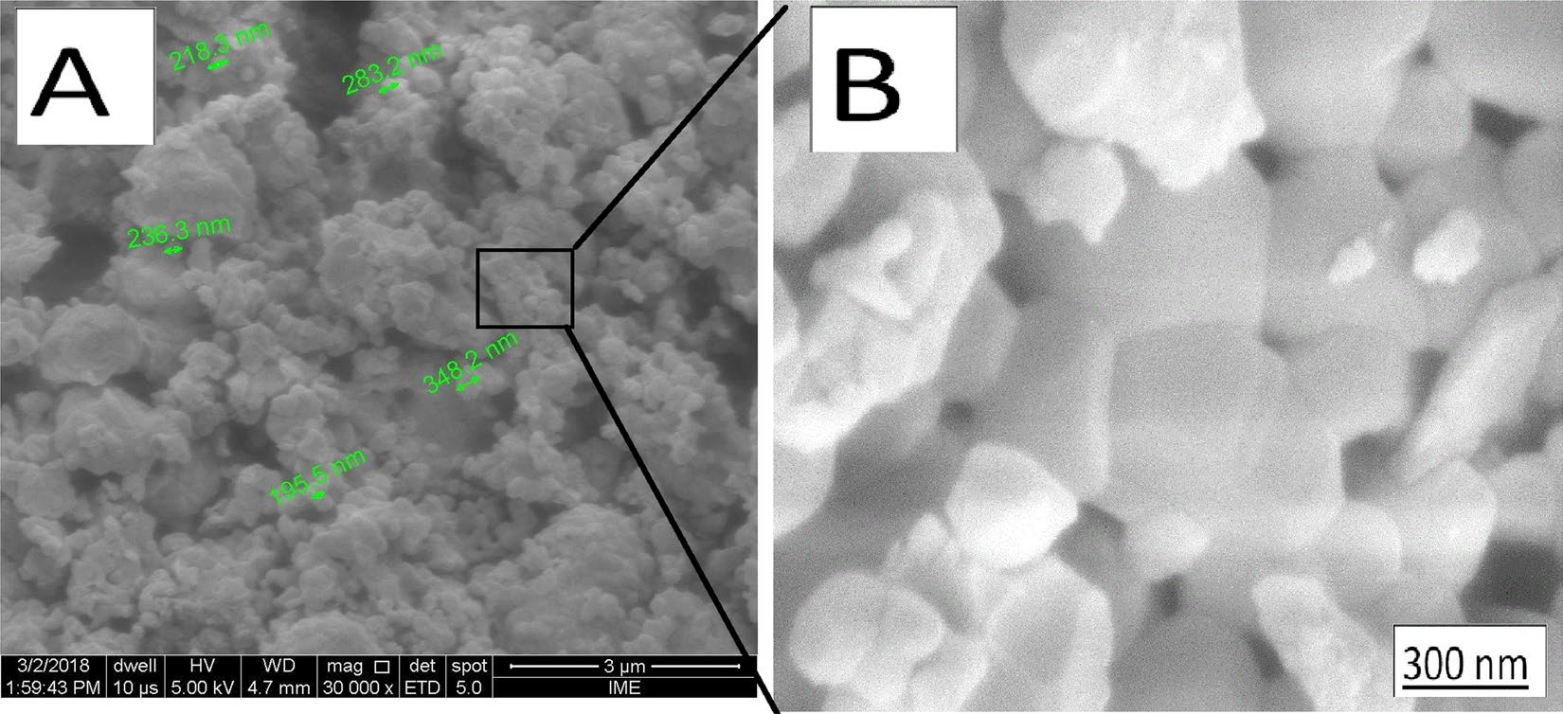
(**A**) Cluster of nanoparticles of Blue Bone biomaterial. (**B**) Details of nanoparticles. It possible to observe nanoporosity in the nanoparticles interface.

Figure 4 shows cluster and nanoparticles of hydroxyapatite.

Cytotoxicity analysis was evaluated by measuring the diameter of the light halo formed around the sample and classified according to the reactivity (Table 2).

**Table 2:** Classification of the cytotoxicity samples by the diameter of the light halo.

Zone of reactivity	Cytotoxicity	Ranking
No detectable zone around or under the sample	None	0
Some malformed or degenerate cells under the sample	Light	1
Zone limited to the area under the sample	Soft	2
Zone extends from 0.5 to 1.0 cm beyond the sample	Moderate	3
Zone extends more than 1.0 cm beyond the sample	Strong	4

The results of the cytotoxicity tests of 20 samples are shown in Table 3. Table 3 shows the cytotoxicity of analyzed biomaterials, the negative surgery site control filled with filter paper and positive surgery site filled with latex fragments. It can be observed that both biomaterials samples showed no evidence of cytotoxicity, since no halo of toxicity was observed around the sample. The cells were intact without any morphological alterations, which were identical characteristics to the negative control. In the positive control, the toxicity characteristic was verified by the presence of a clear halo around the sample, with a diameter of 1.06 mm. This halo is observed when there is lysis and cell death. In this case, the neutral red dye becomes embedded in the cells and gives a transparent appearance to the site, characterizing the toxicity.

**Table 3:** Biomaterials cytotoxicity test results.

Material	Diameter of halo in mm		
	1	2	3
Sample	0	0	0
Negative control	0	0	0
Positive control	1.1	1.0	1.1

Sample is the analyzed biomaterials (Blue Bone and Bio Oss), a negative control is the site filled with filter paper and a positive control is the site filled with latex fragments.

The results of the density measurements determined by helium gas pycnometry are shown in Table 4.

**Table 4:** Samples densities (g/cm^3^) measurements with pycnometry methodology.

Samples	1	2	3	4	5	6	7	8
	3.1310	3.1299	3.1258	3.1226	3.1186	3.1190	3.1045	3.1111
	3.1203 + 0.0091 g/cm^3^							

**Figure 5:**
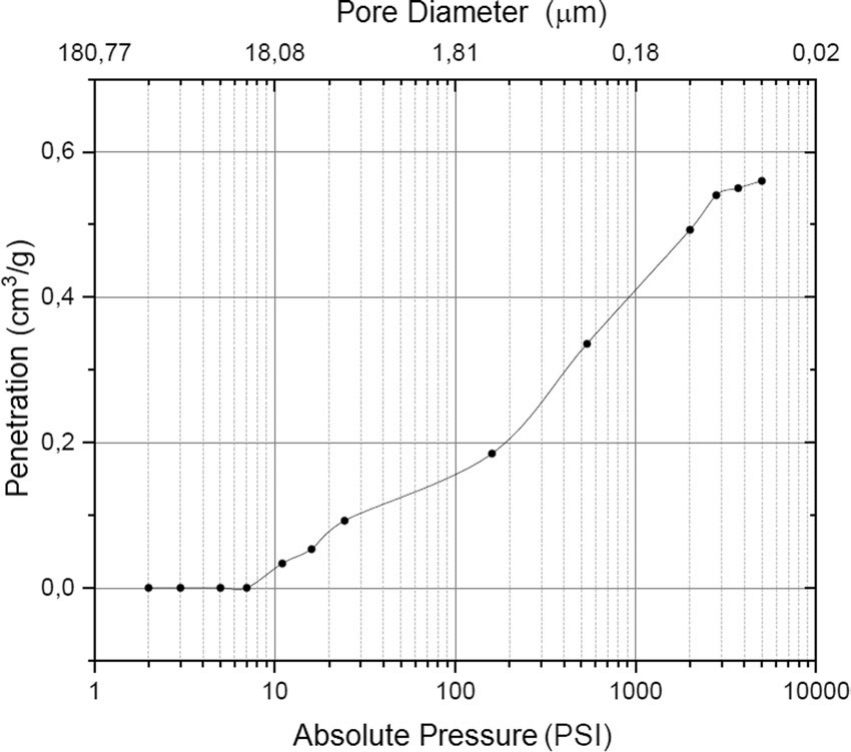
Distribution curve of pore size in the Blue Bone samples, absolute pressure and gas penetration in the biomaterial.

The results obtained in the mercury intrusion porosimetry tests showed that the percentage of total porosity of the Blue Bone samples is 63.84% and pore diameter size is from 0.10 to 18.08 μm. The penetration of gas as a function of absolute pressure is shown in Fig. 5, which shows the pore size distribution.

**Figure 6:**
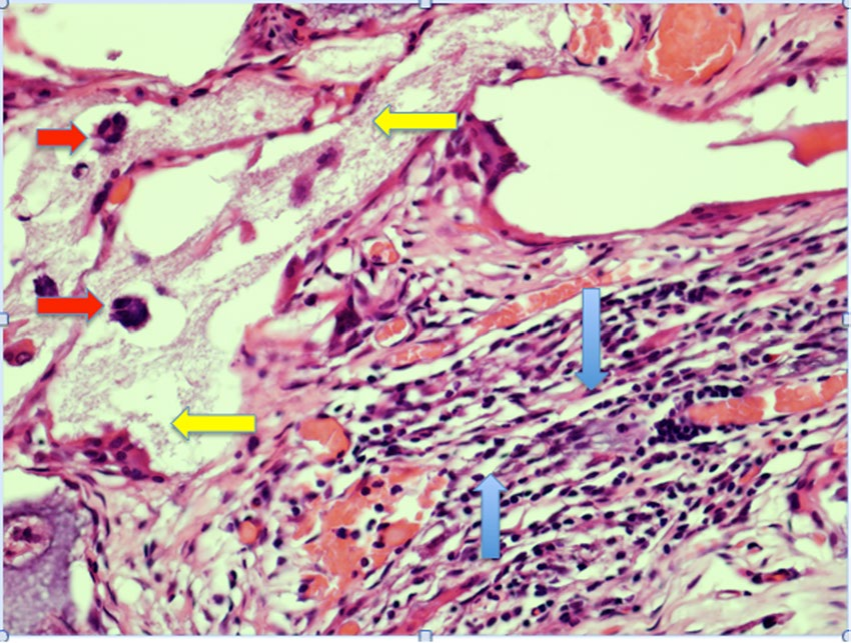
Histomorphometric image of Blue Bone 4 weeks after surgery. Osteoclasts (red arrow), extracellular matrix (yellow arrow) and large migration of cells (blue arrow) were surrounded by the bone marrow. Hematoxylin and eosin stain. 200×, scale bar = 500 µm.

**Figure 7:**
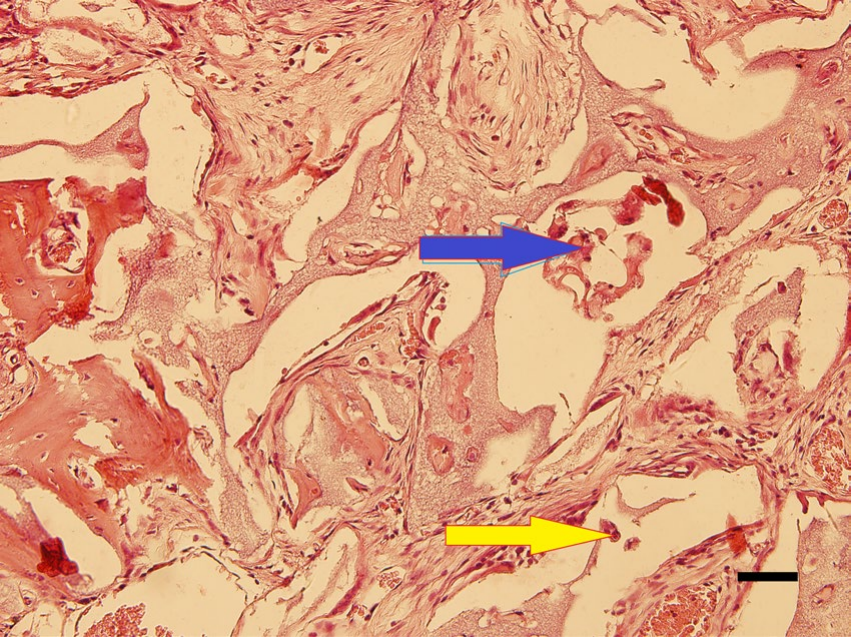
Histomorphometric image of Bio Oss 4 weeks after surgery. Graft demineralized area (yellow arrow), presence of matrix undergoing remodeling process (blue arrow). Hematoxylin and eosin stain. 200×, scale bar = 50 µm.

All surgeries of the experimental groups with Blue Bone and Bio Oss biomaterials were successfully performed. Figures 6 and 7 show the histologic images of the surgery sites. The analyzed biomaterials did not show inflammation or complications at the surgical site. A large bone cell like and a newly formed extracellular matrix were found surrounding Blue Bone particles (Fig. 6).

### Statistics analyze

Based on Figs. 2 and 3, the statistical analysis the following parameters were evaluated: % Graft particle number in the surgery site, % New hard tissue points number, % Graft particle area and % New hard tissue area. The test used was the paired T-test. The following results were obtained (Table 5):

- in the analyzed groups, the coefficient of variation was less than 15%, which means a good representation of the samples, and;
- in the % graft particles area and new tissue area of both biomaterials groups, the significance level (P) was approximately 0.01%, that is, the results are 99.99% certain.

**Table 5:** Histology statistical result of 20 samples analyzed.

	% Biomaterial remaining particles number		% New tissue points number		% Biomaterial remaining particles area		% New tissue area	
	Bio Oss	Blue Bone	Bio Oss	Blue Bone	Bio Oss	Blue Bone	Bio Oss	Blue Bone
P value	0.924		0.4184		0.0012		0.0012	
Average	72.92	72.76	26.9	28.15	31.09	25.12	69.00	74.97
Standard Error	0.5915	1.216	0.577	1.094	0.7318	0.7521	0.6876	0.8082
Coefficient of variation	2.69%	5.54%	7.12%	12.89%	7.81%	9.93%	3.31%	3.58%

% Graft particle number in the surgery site, % New tissue points number, % Graft particle area and % New tissue area. (based on Figs. 2 and 3).

Statistical analysis results showed no significant difference in the number of unabsorbed biomaterial particles between Blue Bone and Bio Oss. The remaining Bio Oss graft granules occupied a higher area than Blue Bone biomaterial. Comparing the percentage of the area occupied by new tissue, the statistical analysis shows that the Blue Bone is higher than Bio Oss.

## Discussion

Hydroxyapatite has good cell conductivity^13^ and allows a good framework for the fibrin network^14^. These characteristics make hydroxyapatite the main synthetic material used in bone grafting surgeries^15^. Hydroxyapatite is used for guided bone reconstruction, along with occlusive barriers, titanium screen, collagen membranes, among other applications^16^.

The characteristics of the synthetic materials used in grafting influence the process of bone remodeling^17^. Particle morphology, crystalline structure, types, percentages of phases, particle size and degradation rate are among the important characteristics. Materials with adequate properties accelerate the mechanisms involved in the process of bone neoformation and increase vascularization in the newly formed bone^18^.

The biomaterial with nanosized particles has a surface area of 30 to 50% greater than biomaterials with micrometric size. The analyzed biomaterial with its nanometric particle size has characteristics that are clinically capable of absorbing a greater volume of fluids when compared with micrometric ones.

The high porosity presented by the synthetic material influences positively the bone remodeling process^18^, increases the wettability of the material and induces the formation of bone with a higher number of blood cells^19^. The results of the present work showed that the analyzed biomaterial presented high levels of porosity with a high percentage of nanometric pores.

According to Kojima *et al*.^20^ the use of composite biomaterials with HA/beta-TCP induced higher cortical bone formation than other biomaterials used as grafting. The explanation is that the rapid reabsorption of beta-TCP promotes better vascularization and induces a more medullary bone formation. On the other side, the low reabsorption of hydroxyapatite gives the regenerated area the hardness needed for more cortical bone formation. Histologically these biomaterials do not present differences in the number of osteocytes in the implantation region when compared to the native bone. Differences are found in the number of osteoclasts and positive mesenchymal cells. The *in vivo* results indicated that biomaterials with HA/beta-TCP composition are suitable for use in the formation of new bone in guided bone regeneration surgery.

A biomaterial should present viable cells around its granules and the newly formed bone matrix must be consistent and homogeneous^21^. In the *in vivo* tests, histological analyses of Blue Bone showed a large number of viable cells around the demineralized granules, in addition to the presence of active osteoclasts, performing the degradation of β-tricalcium phosphate and generating large areas of newly formed bone matrix.

The used of synthesis methodology allows the production of homogeneous hydroxyapatites particle due to great process control. With this, it is possible to obtain different batches of biomaterial having similar properties. This procedure facilitates the adhesion and differentiation of mesenchymal cells. The analyzed biomaterial induces the proliferation of cells related to bone formation. Blue Bone’s manufacturing process is well controlled to allow the manufacture of a biomaterial with homogeneous characteristics and no variation in properties between batches.

The results of the present work corroborate the existing data in the literature, indicating that synthetic hydroxyapatite nanoparticles contribute to the process of bone remodeling. Among the works in the literature^22^ the particle size influences bone formation. The authors observed hydroxyapatite nanoparticles within the phagosome of the analyzed cells. This presence causes reprecipitation of calcium phosphate to prevent the release of large amounts of calcium and phosphate ions into the cells. According to Russmueller^23^, such a phenomenon may be linked to the physiological processes of the cell, which control the concentration and trafficking of intracellular calcium ions, which are highly managed by cells.

The porosity of the biomaterial is essential for the bone conduction process. By moving through the pores of the particles, cells such as osteoclasts and osteoblasts can, together with the inflammatory processes and metalloproteinases, synthesize the hydroxyapatite granules, opening space for the formation of a new bone^24^. Blue Bone has a great potential for bone matrix formation, as it demonstrates *in vivo* tests similarities in all the physicochemical characteristics of the best biomaterials in the market.

The results showed that Blue Bone grafts are an excellent healing material because of its osteoconduction and osteoinduction properties. One advantage of this material is that it can be used without mixing with other bone graft materials. Sample analyses have shown that Blue Bone exhibits similar characteristics to products on the market and may be indicated for all bone regeneration procedures.

## Conclusions

Based on the Blue Bone biomaterial analysis results, it can be concluded that:

- The analyzed composite biomaterial is formed by nano-sized hydroxyapatite with monoclinic symmetry, β-tricalcium phosphate with trigonal structure and CaO with cubic symmetry;
- the biomaterial has 78.76% hydroxyapatite [Ca_5_(PO_4_)_3_(OH)], 21.03% β-tricalcium phosphate [β-Ca_3_(PO_4_)_2_] and 0.19% CaO;
- the characteristics of the composition of the biomaterial are similar to those of the main synthetic products on the market, and should be indicated for all guided bone regeneration procedures;
- the analyzed biomaterial has adequate morphology, zero cytotoxicity, and high porosity;
- the synthetic biomaterial with Blue Bone designation presents characteristics suitable for use in grafting;
- Statistical analyses showed that the Blue Bone induced a higher percentage of new bone tissue than the Bio Oss with an accuracy level of 99.99%.

### Statement

We declare that: (a) The animals were supplied by Centro de Criação de Animais do Instituto de Biologia Roberto Alcantara Gomes (IBRAG, UERJ) Instituto Oswaldo Cruz (Rio de Janeiro, Brazil); (b) This study was approved by the institutional review board and the animal ethical committee at the Oswaldo Cruz Institute; (c) The protocol number was 001-2019 and we confirm that all methods were performed in accordance with the relevant guidelines and regulations.
